# (*Z*)-6-[(5-Chloro-2-hydroxy­phenyl)­aminomethyl­ene]-2-ethoxy­cyclo­hexa-2,4-dienone

**DOI:** 10.1107/S1600536809031298

**Published:** 2009-08-15

**Authors:** Arzu Özek, Çiğdem Albayrak, Orhan Büyükgüngör

**Affiliations:** a Department of Physics, Ondokuz Mayıs University, TR-55139, Samsun, Turkey; b Faculty of Education, Sinop University, Sinop, Turkey

## Abstract

The title compound, C_15_H_14_ClNO_3_, exists as the keto–amine form in the crystal and two intra­molecular N—H⋯O hydrogen bonds are observed. The aromatic rings are oriented at a dihedral angle of 5.85 (8)°. In the crystal structure, inter­molecular O—H⋯O and C—H⋯O hydrogen bonds link the mol­ecules into chains. A π–π contact between the benzene rings [centroid–centroid distance = 3.6623 (10) Å] further stabilizes the structure.

## Related literature

For general background, see: Büyükgüngör *et al.* (2007 ▶); Ersanlı *et al.* (2003 ▶); Tanak *et al.* (2008 ▶) For related structures, see: Özek *et al.* (2007 ▶, 2008 ▶).
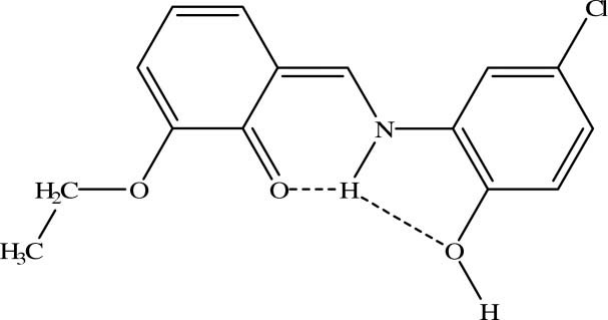


## Experimental

### 

#### Crystal data

C_15_H_14_ClNO_3_
*M* *_r_* = 291.72
Monoclinic, 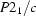

*a* = 15.4313 (7) Å
*b* = 7.1710 (2) Å
*c* = 12.6071 (6) Å
β = 111.168 (4)°
*V* = 1300.94 (10) Å^3^
*Z* = 4
Mo *K*α radiation
μ = 0.30 mm^−1^
*T* = 296 K
0.52 × 0.29 × 0.09 mm

#### Data collection

STOE IPDS II diffractometer
Absorption correction: integration *X-RED32* (Stoe & Cie, 2002 ▶) *T*_min_ = 0.616, *T*_max_ = 0.927
15989 measured reflections
2680 independent reflections
2084 reflections with *I* > 2σ(*I*)
*R*_int_ = 0.060

#### Refinement

*R*[*F* ^2^ > 2σ(*F* ^2^)] = 0.041
*wR*(*F* ^2^) = 0.099
*S* = 1.05
2680 reflections
237 parameters
All H-atom parameters refined
Δρ_max_ = 0.15 e Å^−3^
Δρ_min_ = −0.25 e Å^−3^

### 

Data collection: *X-AREA* (Stoe & Cie, 2002 ▶); cell refinement: *X-AREA*; data reduction: *X-RED32* (Stoe & Cie, 2002 ▶); program(s) used to solve structure: *SHELXS97* (Sheldrick, 2008 ▶); program(s) used to refine structure: *SHELXL97* (Sheldrick, 2008 ▶); molecular graphics: *ORTEP-3 for Windows* (Farrugia, 1997 ▶); software used to prepare material for publication: *WinGX* (Farrugia, 1999 ▶).

## Supplementary Material

Crystal structure: contains datablocks I, global. DOI: 10.1107/S1600536809031298/bt5027sup1.cif

Structure factors: contains datablocks I. DOI: 10.1107/S1600536809031298/bt5027Isup2.hkl

Additional supplementary materials:  crystallographic information; 3D view; checkCIF report

## Figures and Tables

**Table 1:** Hydrogen-bond geometry (Å, °)

*D*—H⋯*A*	*D*—H	H⋯*A*	*D*⋯*A*	*D*—H⋯*A*
N1—H1⋯O1	0.86 (2)	1.88 (2)	2.5957 (17)	140 (2)
N1—H1⋯O3	0.86 (2)	2.29 (2)	2.640 (2)	104.3 (17)
O3—H3⋯O1^i^	0.85 (2)	1.79 (2)	2.6258 (17)	165 (2)
C9—H9⋯O3^ii^	0.96 (2)	2.594 (19)	3.419 (2)	143.8 (14)

Symmetry codes: (i) 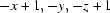; (ii) 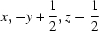.

**Fig. 1.**
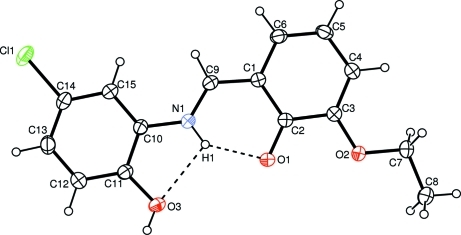
A view of (I), with the atom-numbering scheme and 30% probability displacement ellipsoids. Dashed line indicates intramolecular hydrogen bond.

**Fig. 2.**
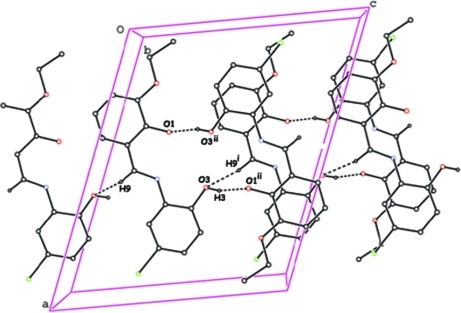
A partial packing view of (I), showing the intermolecular C—H···O and O—H···O hydrogen bonds. Dashed lines indicate hydrogen bonds. H atoms are represented as small spheres of arbitrary radii and H atoms not involved in hydrogen bonding have been omitted for clarity. [Symmetry codes: (i): x, -y + 1/2, z + 1/2; (ii): 1 - x, -y, 1 - z].

## supplementary crystallographic information

### Comment

As part of our ongoing studies on the syntheses and structural characterizations of Schiff-base compounds (Özek *et al.*, 2008; Özek *et al.*, 2007), we report here in the crystal structure of the title compound.

Schiff bases display two possible tautomeric forms, namely phenol-imine (O—H···N) and keto-amine (N—H···O) forms. *o*-Hydroxy Schiff bases have previously been observed in the keto form (Tanak *et al.*, 2008) and in the enol form (Büyükgüngör *et al.*, 2007).

The H atom in title compound (I) is located on atom N1, thus the keto-amine tautomer is favored over the phenol-imine form, as indicated by C2—O1 [1.279 (2) Å], C9—N1 [1.310 (2) Å], C1—C9 [1.410 (2) Å] and C1—C2 [1.432 (2) Å] bonds (Fig. 1). The O1—C2 bond length of 1.279 (2) Å indicates double-bond character, whereas the N1—C9 bond length of 1.310 (2) Å indicates a high degree of single-bond character. Similar results were observed for 2-[(2-Hydroxy- 4-nitrophenyl)-aminomethylene]cyclohexa-3,5-dien-1(2*H*)-οne [C—O = 1.298 (2) and C—N =1.308 (2) Å; Ersanlı *et al.*, 2003].

It is known that Schiff bases may exhibit thermochromism or photochromism, depending on the planarity or non-planarity of the molecule, respectively. Therefore, one can expect thermochromic properties in the title compound caused by planarity of the molecule; the dihedral angle between rings A (C1—C6) and B (C10—C15) is 5.85 (8)°. Intramolecular N—H···O hydrogen bonds (Table 1) result in the formations of planar six- and five-membered rings C (O1/N1/C1/C2/C9/H1) and D (O3/N1/C10/C11/H1). They are oriented with respect to the adjacent rings at dihedral angles of A/C = 1.65 (9), A/D = 5.12 (9), B/C = 7.29 (7), B/D = 4.51 (5) and C/D = 5.61 (12) °. So, they are nearly coplanar.

In the crystal structure, molecules are linked into a three-dimensional network by intermolecular C—H···O and O—H···O hyrogen bonds (Table 1). The C—H···O hydrogen bonds generate C(6) chains along the *c* axis and O—H···O hydrogen bonds generate *R*_2_^2^(18) ring motif (Fig. 2). The π···π contact between the phenyl rings, *Cg*1—*Cg*2^i^ [symmetry code: (i) 1 - *x*, 1/2 + *y*, 1/2 - *z*, where *Cg*1 and *Cg*2 are centroids of the rings A (C1—C6) and B (C10—C15), respectively] may further stabilize the structure, with centroid-centroid distance of 3.6623 (10) Å.

### Experimental

The compound (*Z*)-6-[(5-chloro-2-hydroxyphenylamino)methylene]-2- ethoxycyclohexa-2,4-dienone was prepared by reflux a mixture of a solution containing 3-ethoxy-2-hydroxybenzaldehyde (0.5 g 3 mmol) in 20 ml e thanol and a solution containing 5-chloro-2-hydroxyaniline (0.43 g 3 mmol) in 20 ml e thanol. The reaction mixture was stirred for 1 h under reflux. The crystals of (*Z*)-6-[(5-chloro-2-hydroxyphenylamino) methylene]-2-ethoxycyclohexa-2,4-dienone suitable for X-ray analysis were obtained from ethanol by slow evaporation (yield % 89; m.p. 489–491 K).

### Refinement

H atoms were located in difference synthesis, and refined freely.

### Crystal data

**Table:**

C_15_H_14_ClNO_3_	*F*(000) = 608
*M**_r_* = 291.72	*D*_x_ = 1.489 Mg m^−^^3^
Monoclinic, *P*2_1_/*c*	Mo *K*α radiation, λ = 0.71073 Å
Hall symbol: -P 2ybc	Cell parameters from 15989 reflections
*a* = 15.4313 (7) Å	θ = 2.8–26.5°
*b* = 7.1710 (2) Å	µ = 0.30 mm^−^^1^
*c* = 12.6071 (6) Å	*T* = 296 K
β = 111.168 (4)°	Prism, red
*V* = 1300.94 (10) Å^3^	0.52 × 0.29 × 0.09 mm
*Z* = 4	

### Data collection

**Table:**

STOE IPDS II diffractometer	2680 independent reflections
Radiation source: fine-focus sealed tube	2084 reflections with *I* > 2σ(*I*)
plane graphite	*R*_int_ = 0.060
Detector resolution: 6.67 pixels mm^-1^	θ_max_ = 26.5°, θ_min_ = 2.8°
ω scans	*h* = −19→19
Absorption correction: integration *X-RED32* (Stoe & Cie, 2002)	*k* = −8→8
*T*_min_ = 0.616, *T*_max_ = 0.927	*l* = −15→15
15989 measured reflections	

### Refinement

**Table:**

Refinement on *F*^2^	Primary atom site location: structure-invariant direct methods
Least-squares matrix: full	Secondary atom site location: difference Fourier map
*R*[*F*^2^ > 2σ(*F*^2^)] = 0.041	Hydrogen site location: inferred from neighbouring sites
*wR*(*F*^2^) = 0.099	All H-atom parameters refined
*S* = 1.05	*w* = 1/[σ^2^(*F*_o_^2^) + (0.0546*P*)^2^ + 0.0402*P*] where *P* = (*F*_o_^2^ + 2*F*_c_^2^)/3
2680 reflections	(Δ/σ)_max_ < 0.001
237 parameters	Δρ_max_ = 0.14 e Å^−^^3^
0 restraints	Δρ_min_ = −0.25 e Å^−^^3^

### Special details

**Table:**

Experimental. 314 frames, detector distance = 100 mm
Geometry. All e.s.d.'s (except the e.s.d. in the dihedral angle between two l.s. planes) are estimated using the full covariance matrix. The cell e.s.d.'s are taken into account individually in the estimation of e.s.d.'s in distances, angles and torsion angles; correlations between e.s.d.'s in cell parameters are only used when they are defined by crystal symmetry. An approximate (isotropic) treatment of cell e.s.d.'s is used for estimating e.s.d.'s involving l.s. planes.
Refinement. Refinement of *F*^2^ against ALL reflections. The weighted *R*-factor *wR* and goodness of fit *S* are based on *F*^2^, conventional *R*-factors *R* are based on *F*, with *F* set to zero for negative *F*^2^. The threshold expression of *F*^2^ > σ(*F*^2^) is used only for calculating *R*-factors(gt) *etc*. and is not relevant to the choice of reflections for refinement. *R*-factors based on *F*^2^ are statistically about twice as large as those based on *F*, and *R*- factors based on ALL data will be even larger.

### Fractional atomic coordinates and isotropic or equivalent isotropic displacement parameters (Å^2^)

**Table:**

	*x*	*y*	*z*	*U*_iso_*/*U*_eq_	
C1	0.42045 (11)	0.1210 (2)	0.12588 (13)	0.0348 (3)	
C2	0.35762 (11)	0.0951 (2)	0.18490 (13)	0.0347 (3)	
C3	0.25975 (11)	0.0968 (2)	0.11617 (14)	0.0354 (4)	
C4	0.23136 (12)	0.1153 (2)	0.00038 (14)	0.0405 (4)	
C5	0.29497 (13)	0.1391 (3)	−0.05529 (15)	0.0420 (4)	
C6	0.38735 (12)	0.1431 (2)	0.00582 (14)	0.0397 (4)	
C7	0.10537 (12)	0.1023 (3)	0.11398 (16)	0.0429 (4)	
C8	0.05590 (13)	0.1030 (4)	0.19698 (19)	0.0515 (5)	
C9	0.51704 (11)	0.1302 (2)	0.18651 (15)	0.0372 (4)	
C10	0.65028 (11)	0.1159 (2)	0.36438 (14)	0.0349 (3)	
C11	0.67292 (11)	0.1056 (2)	0.48215 (14)	0.0366 (4)	
C12	0.76552 (12)	0.0967 (3)	0.55291 (16)	0.0435 (4)	
C13	0.83466 (12)	0.0983 (3)	0.50765 (16)	0.0446 (4)	
C14	0.81081 (11)	0.1101 (2)	0.39143 (16)	0.0398 (4)	
C15	0.71950 (12)	0.1205 (2)	0.31875 (15)	0.0385 (4)	
N1	0.55488 (9)	0.1157 (2)	0.29736 (12)	0.0375 (3)	
O1	0.38649 (8)	0.07391 (19)	0.29284 (10)	0.0458 (3)	
O2	0.20269 (8)	0.08261 (18)	0.17686 (10)	0.0432 (3)	
O3	0.60157 (9)	0.10408 (19)	0.52038 (11)	0.0462 (3)	
Cl1	0.89826 (3)	0.10870 (7)	0.33413 (5)	0.05476 (17)	
H1	0.5154 (16)	0.095 (3)	0.3294 (19)	0.057 (6)*	
H3	0.6156 (16)	0.048 (3)	0.584 (2)	0.058 (6)*	
H4	0.1662 (17)	0.112 (3)	−0.044 (2)	0.067 (7)*	
H5	0.2700 (13)	0.157 (3)	−0.1342 (18)	0.048 (5)*	
H6	0.4309 (14)	0.169 (3)	−0.0299 (16)	0.047 (5)*	
H7A	0.0925 (14)	0.221 (3)	0.0713 (18)	0.055 (6)*	
H7B	0.0851 (14)	−0.005 (3)	0.0600 (18)	0.049 (5)*	
H8A	0.0771 (17)	0.197 (3)	0.250 (2)	0.067 (7)*	
H8B	0.0646 (15)	−0.015 (4)	0.2363 (19)	0.063 (6)*	
H8C	−0.0076 (18)	0.120 (3)	0.157 (2)	0.068 (7)*	
H9	0.5538 (13)	0.150 (2)	0.1399 (16)	0.041 (5)*	
H12	0.7798 (15)	0.087 (3)	0.633 (2)	0.055 (6)*	
H13	0.8974 (15)	0.090 (3)	0.5585 (18)	0.053 (6)*	
H15	0.7051 (13)	0.124 (3)	0.2410 (18)	0.046 (5)*	

### Atomic displacement parameters (Å^2^)

**Table:**

	*U*^11^	*U*^22^	*U*^33^	*U*^12^	*U*^13^	*U*^23^
C1	0.0326 (8)	0.0412 (8)	0.0316 (8)	0.0001 (7)	0.0127 (6)	−0.0005 (7)
C2	0.0332 (8)	0.0416 (8)	0.0302 (8)	0.0009 (7)	0.0124 (6)	0.0021 (6)
C3	0.0329 (8)	0.0404 (8)	0.0331 (8)	−0.0007 (7)	0.0122 (6)	−0.0005 (7)
C4	0.0356 (8)	0.0490 (9)	0.0333 (8)	0.0003 (7)	0.0081 (7)	−0.0003 (7)
C5	0.0454 (10)	0.0520 (11)	0.0264 (8)	0.0044 (8)	0.0103 (7)	0.0021 (7)
C6	0.0425 (9)	0.0470 (10)	0.0344 (9)	0.0034 (7)	0.0197 (7)	0.0042 (7)
C7	0.0294 (8)	0.0547 (11)	0.0421 (9)	0.0028 (8)	0.0099 (7)	0.0023 (9)
C8	0.0334 (9)	0.0712 (14)	0.0510 (11)	0.0059 (9)	0.0167 (9)	0.0038 (11)
C9	0.0349 (8)	0.0439 (9)	0.0368 (8)	−0.0001 (7)	0.0178 (7)	0.0027 (7)
C10	0.0298 (7)	0.0389 (8)	0.0367 (9)	−0.0004 (6)	0.0129 (6)	0.0021 (7)
C11	0.0352 (8)	0.0407 (8)	0.0373 (8)	0.0004 (7)	0.0172 (7)	0.0021 (7)
C12	0.0382 (9)	0.0533 (10)	0.0365 (9)	0.0016 (8)	0.0102 (7)	0.0024 (8)
C13	0.0312 (8)	0.0510 (10)	0.0475 (10)	0.0006 (7)	0.0091 (7)	0.0016 (8)
C14	0.0313 (8)	0.0409 (9)	0.0511 (10)	−0.0009 (7)	0.0197 (7)	0.0017 (8)
C15	0.0361 (8)	0.0447 (9)	0.0383 (9)	0.0008 (7)	0.0178 (7)	0.0007 (7)
N1	0.0295 (7)	0.0513 (8)	0.0334 (7)	−0.0013 (6)	0.0136 (6)	0.0017 (6)
O1	0.0332 (6)	0.0767 (9)	0.0287 (6)	−0.0018 (6)	0.0125 (5)	0.0055 (6)
O2	0.0277 (6)	0.0683 (8)	0.0333 (6)	−0.0003 (5)	0.0107 (5)	0.0035 (5)
O3	0.0403 (7)	0.0646 (8)	0.0403 (7)	0.0069 (6)	0.0225 (6)	0.0103 (6)
Cl1	0.0386 (2)	0.0643 (3)	0.0721 (3)	0.0028 (2)	0.0328 (2)	0.0067 (2)

### Geometric parameters (Å, °)

**Table:**

C1—C9	1.410 (2)	C8—H8C	0.93 (3)
C1—C6	1.421 (2)	C9—N1	1.310 (2)
C1—C2	1.432 (2)	C9—H9	0.96 (2)
C2—O1	1.279 (2)	C10—C15	1.384 (2)
C2—C3	1.445 (2)	C10—C11	1.399 (2)
C3—O2	1.363 (2)	C10—N1	1.408 (2)
C3—C4	1.371 (2)	C11—O3	1.3519 (19)
C4—C5	1.408 (3)	C11—C12	1.386 (2)
C4—H4	0.96 (2)	C12—C13	1.380 (3)
C5—C6	1.354 (2)	C12—H12	0.95 (2)
C5—H5	0.94 (2)	C13—C14	1.378 (3)
C6—H6	0.95 (2)	C13—H13	0.95 (2)
C7—O2	1.430 (2)	C14—C15	1.378 (2)
C7—C8	1.502 (3)	C14—Cl1	1.7456 (17)
C7—H7A	0.99 (2)	C15—H15	0.92 (2)
C7—H7B	1.00 (2)	N1—H1	0.86 (2)
C8—H8A	0.92 (2)	O3—H3	0.85 (2)
C8—H8B	0.97 (2)		
			
C9—C1—C6	118.39 (15)	H8A—C8—H8C	109 (2)
C9—C1—C2	120.41 (15)	H8B—C8—H8C	108.4 (19)
C6—C1—C2	121.18 (14)	N1—C9—C1	123.47 (15)
O1—C2—C1	121.83 (14)	N1—C9—H9	121.9 (11)
O1—C2—C3	121.77 (14)	C1—C9—H9	114.6 (11)
C1—C2—C3	116.40 (14)	C15—C10—C11	120.52 (15)
O2—C3—C4	125.61 (15)	C15—C10—N1	123.16 (15)
O2—C3—C2	114.20 (14)	C11—C10—N1	116.29 (14)
C4—C3—C2	120.18 (15)	O3—C11—C12	123.59 (16)
C3—C4—C5	122.02 (16)	O3—C11—C10	117.09 (15)
C3—C4—H4	118.9 (14)	C12—C11—C10	119.32 (15)
C5—C4—H4	119.0 (14)	C13—C12—C11	120.32 (17)
C6—C5—C4	119.94 (16)	C13—C12—H12	121.3 (13)
C6—C5—H5	123.1 (12)	C11—C12—H12	118.4 (13)
C4—C5—H5	116.9 (12)	C14—C13—C12	119.38 (16)
C5—C6—C1	120.23 (16)	C14—C13—H13	122.5 (13)
C5—C6—H6	120.8 (12)	C12—C13—H13	118.1 (13)
C1—C6—H6	118.8 (12)	C15—C14—C13	121.78 (16)
O2—C7—C8	108.09 (15)	C15—C14—Cl1	118.87 (14)
O2—C7—H7A	110.6 (12)	C13—C14—Cl1	119.34 (13)
C8—C7—H7A	108.8 (12)	C14—C15—C10	118.66 (16)
O2—C7—H7B	108.1 (12)	C14—C15—H15	120.2 (12)
C8—C7—H7B	111.4 (12)	C10—C15—H15	121.0 (12)
H7A—C7—H7B	109.8 (17)	C9—N1—C10	127.35 (15)
C7—C8—H8A	111.7 (15)	C9—N1—H1	113.4 (15)
C7—C8—H8B	110.1 (14)	C10—N1—H1	119.0 (15)
H8A—C8—H8B	109 (2)	C3—O2—C7	116.33 (13)
C7—C8—H8C	109.0 (15)	C11—O3—H3	112.0 (16)
			
C9—C1—C2—O1	−2.2 (3)	C15—C10—C11—C12	−1.1 (3)
C6—C1—C2—O1	179.60 (16)	N1—C10—C11—C12	177.13 (16)
C9—C1—C2—C3	176.96 (15)	O3—C11—C12—C13	179.80 (17)
C6—C1—C2—C3	−1.3 (2)	C10—C11—C12—C13	0.1 (3)
O1—C2—C3—O2	2.8 (2)	C11—C12—C13—C14	0.5 (3)
C1—C2—C3—O2	−176.34 (14)	C12—C13—C14—C15	0.0 (3)
O1—C2—C3—C4	−178.42 (16)	C12—C13—C14—Cl1	−179.07 (14)
C1—C2—C3—C4	2.5 (2)	C13—C14—C15—C10	−1.0 (3)
O2—C3—C4—C5	176.54 (16)	Cl1—C14—C15—C10	178.08 (13)
C2—C3—C4—C5	−2.1 (3)	C11—C10—C15—C14	1.5 (3)
C3—C4—C5—C6	0.4 (3)	N1—C10—C15—C14	−176.58 (16)
C4—C5—C6—C1	0.8 (3)	C1—C9—N1—C10	177.28 (17)
C9—C1—C6—C5	−178.59 (17)	C15—C10—N1—C9	−4.2 (3)
C2—C1—C6—C5	−0.3 (3)	C11—C10—N1—C9	177.63 (17)
C6—C1—C9—N1	178.49 (16)	C4—C3—O2—C7	−4.7 (2)
C2—C1—C9—N1	0.2 (3)	C2—C3—O2—C7	174.00 (15)
C15—C10—C11—O3	179.17 (15)	C8—C7—O2—C3	−174.00 (16)
N1—C10—C11—O3	−2.6 (2)		

### Hydrogen-bond geometry (Å, °)

**Table:**

*D*—H···*A*	*D*—H	H···*A*	*D*···*A*	*D*—H···*A*
N1—H1···O1	0.86 (2)	1.88 (2)	2.5957 (17)	140 (2)
N1—H1···O3	0.86 (2)	2.29 (2)	2.640 (2)	104.3 (17)
O3—H3···O1^i^	0.85 (2)	1.79 (2)	2.6258 (17)	165 (2)
C9—H9···O3^ii^	0.96 (2)	2.594 (19)	3.419 (2)	143.8 (14)

Symmetry codes: (i) −*x*+1, −*y*, −*z*+1; (ii) *x*, −*y*+1/2, *z*−1/2.
